# Assessing floppy infants: a new module

**DOI:** 10.1007/s00431-022-04476-x

**Published:** 2022-05-04

**Authors:** Costanza Cutrona, Elisa Pede, Roberto De Sanctis, Giorgia Coratti, Eloisa Tiberi, Rita Luciano, Maria Carmela Pera, Chiara Velli, Anna Capasso, Giovanni Vento, Domenico M. Romeo, Marika Pane, Eugenio Mercuri

**Affiliations:** 1 Centro Clinico Nemo Pediatrico, Fondazione Policlinico “A. Gemelli” IRCCS, Rome, Italy; 2 Pediatric Neurology Unit, Università Cattolica del Sacro Cuore, Rome, Italy; 3 Neonatology Unit, Fondazione Policlinico Universitario “A. Gemelli” IRCCS, Rome, Italy; 4 Neonatology Unit, Università Cattolica del Sacro Cuore, Rome, Italy

**Keywords:** Neonate, Hypotonia, Weakness, Floppy, Examination

## Abstract

Our aim was to develop a new module for assessing the floppy infant, to describe the application of the module in a cohort of low-risk newborns and piloting the module in a cohort of floppy infants. The module was applied to a cohort of 143 low-risk newborns and piloted in in a cohort of 24 floppy infants. The new add-on module includes a neurological section and provides a section for recording information obtained by physical examination and antenatal history. For each item, column 1 reports abnormal findings, column 3 normal findings, and column 2 intermediate signs to be followed. Consistent with previous studies, in low-risk infants, none had definitely abnormal or mildly abnormal signs, with the exception of tendon reflexes that were not easily elicitable in 17.14% of term-born infants.

*Conclusion*: Our study suggest that the module can be easily used in a clinical setting as an add-on to the regular neonatal neurological examination in newborns identified as hypotonic on routine examination. Larger cohorts are needed to establish the accuracy of the prognostic value of the module in the differential diagnosis of floppy infant.

**Table:** 

**What is Known:** *• Hypotonia is one of the key signs in newborns with neuromuscular disorders and can be associated with a wide range of other conditions (central nervous system involvement, genetic and metabolic diseases).* *• Weakness or/and contractures can identify infants with a neuromuscular disorder and help in the differential diagnosis of floppy infants.*	
**What is New:** *• To date, this is the first attempt to develop and apply a specific neurological module for the assessment of the floppy infant.* *• The module can be used in a routine clinical setting as an add-on to the regular neurological examination and has potential to differentiate the floppy infants from the low-risk infants.*

**What is Known:**

*• Hypotonia is one of the key signs in newborns with neuromuscular disorders and can be associated with a wide range of other conditions (central nervous system involvement, genetic and metabolic diseases).*

*• Weakness or/and contractures can identify infants with a neuromuscular disorder and help in the differential diagnosis of floppy infants.*

**What is New:**

*• To date, this is the first attempt to develop and apply a specific neurological module for the assessment of the floppy infant.*

*• The module can be used in a routine clinical setting as an add-on to the regular neurological examination and has potential to differentiate the floppy infants from the low-risk infants.*

## Introduction

Generalized hypotonia is a relatively common finding in newborns [1–3]. It manifests as decreased muscle tone that often affects both trunks and limbs, combined with increased popliteal angles, scarf signs, and poor axial tone. In 1968, Victor Dubowitz reported the description of a floppy infant, suggesting that the definition of floppy infant should be based on clinical examination [1]. The criteria include generalized hypotonia, as the infant does not have the normal flexor tone observed in healthy newborns, lax ligaments (excessive range of joint mobility), and on observation, the infant with hypotonia “looks” floppy, with the appearance resembling a “rag doll,” especially when held in ventral suspension. Over the years, there has been increasing attention paid to the clinical signs associated with hypotonia in floppy infants as hypotonia is one of the key signs in newborns with neuromuscular disorders but can also be found in other conditions. An accurate differential diagnosis is therefore needed to ascertain whether the hypotonia is related to central nervous system (CNS) involvement, and genetic or metabolic diseases [4–8].

In the original description, Dubowitz reported that some clinical signs, namely weakness or contractures, can identify infants with a neuromuscular disorder and may help in the differential diagnosis [1]. This has been further confirmed by more recent studies using a systematic approach, reporting that the sensitivity of weakness and contractures to identify peripheral involvement was of 0.97 and 0.69 respectively [1, 2]. Other conditions could, in contrast, be suspected from the absence of weakness and the presence of other clinical signs. Reduced visual alertness, convulsions, or differential tone patterns were more often associated with central nervous system involvement while dysmorphic features and other organ involvement were generally found in genetic or metabolic disorders. A detailed family and antenatal history were often also useful in the differential diagnosis.

Recent advances in the field of neuromuscular disorders and neonatal neurology have completely changed both the diagnostic pathway and the level of intervention possible; but despite the tremendous advances in imaging and in genetic diagnostic tools such as next-generation sequencing (NGS) or whole-exome sequencing [7, 9–12], there is still the need to perform a detailed clinical assessment for targeting and for the interpretation of the genetic findings. This is particularly true considering that at global level, the up-to-date genetic investigations are not always available or there may be a significant delay until the results of genetic tests are received.

Although most clinical neonatal neurological examinations include the assessment of tone, movements, and many other clinical signs that can be associated with hypotonia, there has been no effort to develop a structured form including all these aspects in a format that could help the clinician in the differential diagnosis. This is partly due to the fact that the existing neurological assessments are already perceived as time-consuming and difficult to be used in routine clinical practice. Because of this, we aimed to develop a form that should be used as an add-on module only in the cases where the neurological examination shows obvious signs of hypotonia and therefore does not affect the length of the routine clinical examination.

In this paper, we report the development of a module designed to record different clinical features often associated with hypotonia that could be easily used in a clinical setting. More specifically, we wished to describe the development of the scale, and its application in both low-risk infants and in those with neonatal hypotonia.

## Material and methods

### Development of the module

Our aim was to develop a module that could be easily used in clinical practice and capture a number of aspects known to be associated with neonatal hypotonia.

The selection of items was based on our clinical experience and on criteria previously used to select meaningful clinical features associated with hypotonia [2, 3, 6].

The format used for the new module was similar to that used for the Hammersmith Neonatal Neurological Examination (HNNE) [13]. The assessment findings for each item can be documented on the form in one of three columns. Based on our previous study reporting the frequency distribution of abnormal findings, we designed the form so that the third column includes the findings that are considered optimal, the first column items that are known to be often abnormal, and the second column intermediate findings that require surveillance.

### Application to low-risk newborns

In order to verify that the items in columns 1 and 2 do not generally occur in low-risk infants, the module was applied to a cohort of 143 low-risk newborns including term-born, near-term, and preterm infants (GA ≥ 37, ≥ 34– < 37, and < 34 weeks respectively) routinely followed in our Unit. Infants were defined as low risk in the absence of abnormal neurological signs and, in preterm infants in whom cranial ultrasound scans are performed routinely, also the absence of major changes such as large hemorrhages (grades II–IV), evidence of severe white matter injury, or other clearly abnormal findings. The presence of minor changes such as transient periventricular echodensities and germinal matrix hemorrhages (grade I) was not considered the exclusion criteria.

The new add-on module was performed by 3 examiners during the pre-discharge neonatal examination (between 24 and 48 h of age) for term and late preterm infants, and in the Neonatal Intensive Care Unit for preterm infants. Preterm infants were examined both in the first week after birth and at term age.

### Pilot application in floppy infants

The new module was also prospectively piloted in all the newborns with generalized hypotonia referred or admitted in our Unit since 2016.

The study was approved by the Ethics committee (prot. N. 003,475/21).

### Statistical analysis

Demographic and clinical characteristics were summarized using frequencies (percentage) for categorical variables and mean (standard deviation (SD)). Inter-observer reliability was determined using intra-class correlation coefficients (ICC) and 95% confidence intervals (CI) with a 2-way random effects, single measures, analysis of variance model.

## Results

### Development of the module

The new add-on module includes a neurological section with items that are not included in the standard HNNE but also provides a section for recording information, obtained by physical examination or antenatal history, that are also relevant for the differential diagnosis of the floppy infant.

#### Neurological section

**Fig. 1:**
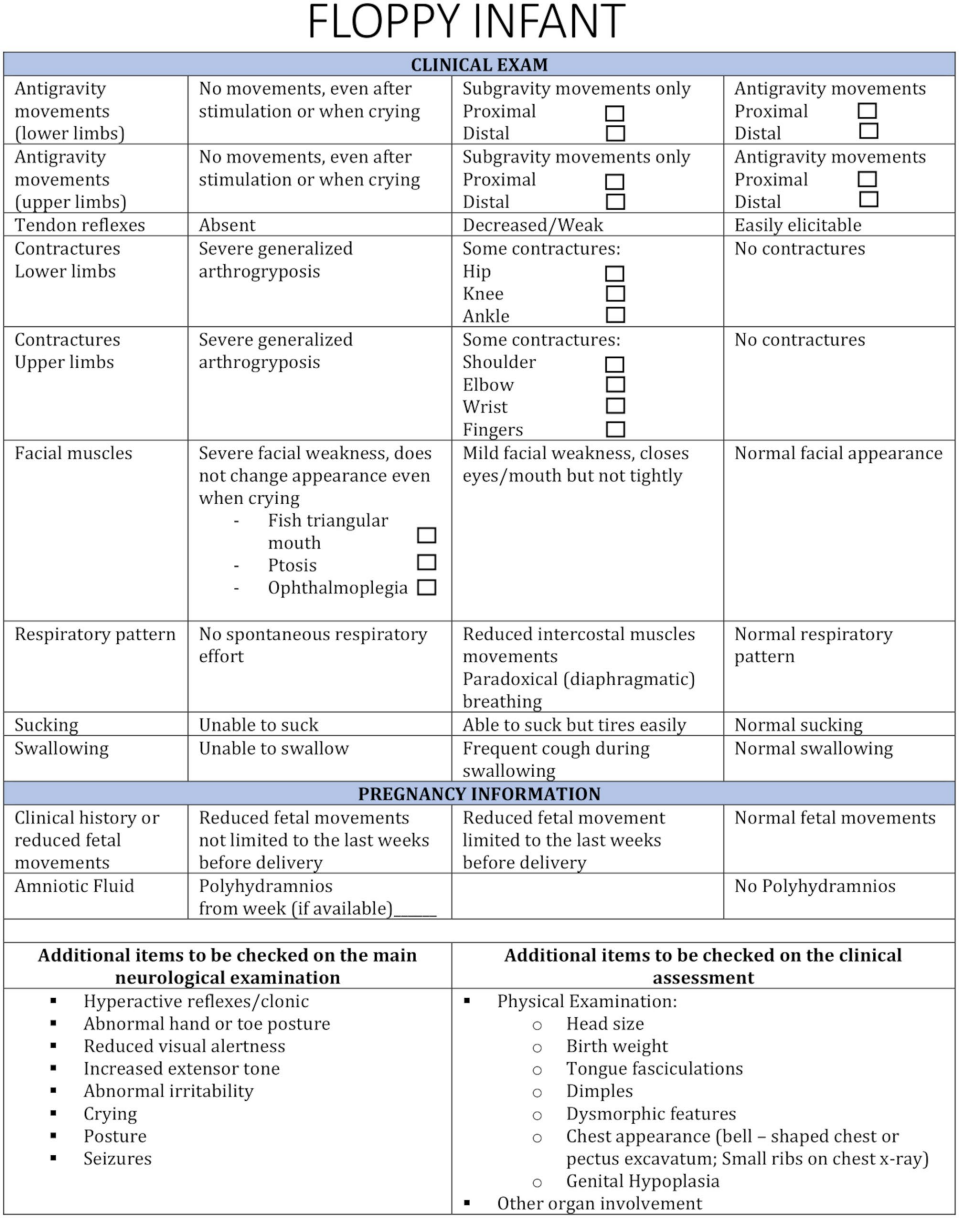
Proforma for the assessment of the floppy infant

This includes 9 new items based on our experience and from the literature that we consider the most relevant for the purpose of our add-on module (Fig. 1).

The first two items assess antigravity movements in the upper and lower limbs as markers of weakness. Weak infants are unable or have difficulties in performing antigravity movements even in response to stimulation. If no spontaneous movement is observed when the infants is awake and calm, it is important also to assess the presence of movements at peak of activity, such as when crying or stimulated.

Item number 3 explores tendon reflexes ranging from easily elicitable to absent, providing more details than the item in the HNNE. The reflexes that are routinely examined are biceps, patella, and Achilles tendon reflexes.

Items 4 and 5 assess contractures in both upper and lower limbs. Neonatal contractures are often the result of reduced motility in utero. We included assessment of possible contractures, especially at the ankle, knee, hip shoulder, and elbow level, where they can only be fully observed by performing an assessment of each joint looking if there is a full range of extension.

Item 6 assesses facial muscle weakness also providing the clue to look for specific aspects of oral or ocular weakness, such as ptosis and fish triangular mouth that are known to be associated with myopathies, myasthenia, and other disorders.

Items 7 and 8 assess sucking and swallowing in more details than assessed in the standard HNNE.

Item number 9 assesses the respiratory pattern, as the selective involvement of diaphragmatic or intercostal muscles can also be a sign of peripheral muscle involvement.

The neurological section also provides a reference to other neurological items, already recorded as part of the main HNNE, including visual alertness, differential tone patterns, and abnormal postures, that are more suggestive of central nervous system involvement and should also be considered in the differential diagnosis of the floppy infant.

#### Prenatal findings

This includes two items: report of poor fetal movements and polyhydramnios suggesting prenatal involvement of the swallowing muscles.

#### Additional items to be checked on the main neurological examinations

In order to avoid repetition with the main neurological examination, a number of items that are potentially relevant for the differential diagnosis of the floppy infant are listed so that could be used, in combination with the new items for diagnostic purposes.

#### Physical examination

This section provides a list of physical findings that are known to be associated with genetic (craniofacial and body dysmorphisms, other organ involvement, birth weight, height and head circumference, and centile) or metabolic disorders (other organ involvement) or to other signs that are only found in specific neuromuscular disorders (e.g., tongue fasciculations,).

#### Inter-observer reliability

The ICC (2,1) was 0.947 (*n* = 3, *p* < 0.001).

### Application to low-risk newborns

The module was applied to 143 low-risk newborns (100 term-born or late preterm and 43 preterm). The gestational age of the preterm infants ranged between 25 and 34 weeks. All the examined infants had scores in column 3 on all the items with the exception of the item assessing reflexes. Not easily elicitable reflexes (column 2) were found in 17% of term-born and late preterm newborns and in 4.7% of preterm infants. Table 1 gives details of the characteristics of the two populations.

**Table 1:** Characteristics of the cohort studied

	**Term and late preterm newborns (*n* = 100)**	**Preterm newborns (*n* = 43)**
Male n (%)	42 (42%)	18 (42%)
Female, *n* (%)	58 (58%)	25 (58%)
Gestational age (GA), *n* (%)	≥ 37 wk: 92 (92%) 35–36 wk: 8 (8%)	< 28 wk: 10 (23%) 28–32 wk: 30 (70%) 33–34 wk: 3 (7%)
Birth weight for GA (mean grams — SD)	≥ 37 wk: 2941 (± 455) 35–36 wk: 2503 (± 182)	< 28 wk: 953 (± 152,027) 28–32 wk: 1274.77 (± 379.233) 33–34 wk: 2240 (± 560.446)
5′ Apgar for GA (mean points — SD)	≥ 37 wk: 9.75 (± 0.46) 35–36 wk: 9.78 (± 0.49)	< 28 wk: 7.75 (± 1.04) 28–32 wk: 8.42 (± 0.81) 33–34 wk: 8.33 (± 0.58)

### Pilot application in floppy infants

The new module was piloted in 24 infants born in or referred to our unit since the module was developed. Table 2 shows details of the findings according to the final diagnosis made.

**Table 2:**
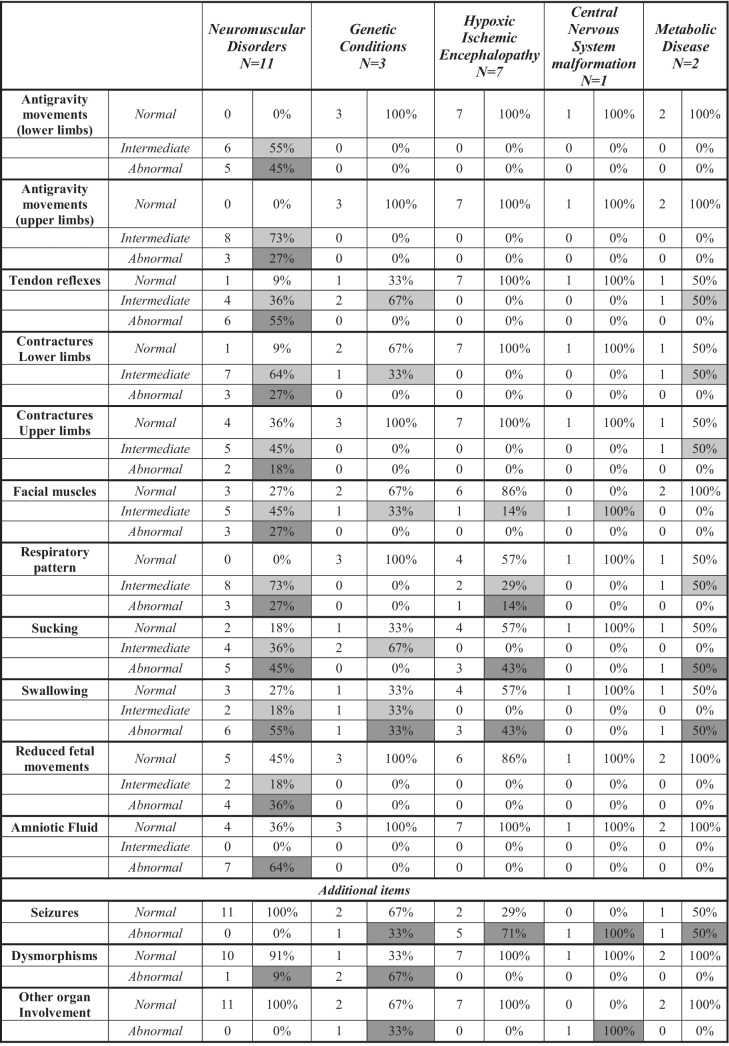
Frequency distribution of findings on the floppy infant module in different clinical subgroups. Key to table: light grey= intermediate scores findings, dark grey: abnormal scores findings

Antigravity movements were abnormal in 11 of the 24 infants: all these infants had a diagnosis of a neuromuscular disorder. Twelve infants had contractures: 10/12 had a diagnosis of a neuromuscular disorder, one had a genetic condition, and one had a metabolic disorder. Other items, such as respiratory patterns and sucking and swallowing abnormalities, were frequently abnormal in different diagnostic groups. Seizures were frequent in disorders affecting the CNS and in metabolic disorders. Dysmorphic features were frequent in genetic disorders.

## Discussion

Our experience suggests that the newly developed module for assessing the floppy infant can be easily administered in a short time. The module was always completed within 2 to 3 min and there was excellent inter-observer reliability. The choice of items was based on the identification of neurological findings that in previous systematic studies and in our clinical experience had the ability to help in the differential diagnosis [2, 3, 6]. We initially included items useful in detecting abnormal signs suggesting peripheral nervous system involvement. We also added other items that would not only help in identifying a neuromuscular problem but may also provide additional information on specific diseases or groups of diseases within the spectrum of neuromuscular disorders. Facial weakness, for example, is invariably found in myotonic dystrophy and in several congenital myopathies or muscular dystrophies while is never observed in spinal muscular atrophy (SMA) [8]. The involvement of respiratory muscles, both as intercostal and diaphragmatic weakness, was also recorded as these are frequent in several neuromuscular disorders with neonatal onset and can help in the differential diagnosis. Other items, such as reflexes or visual attention, were added to identify possible CNS involvement or metabolic or genetic disorders.

The application of the module to a low-risk cohort confirmed that all the items showed normal responses with the absence of signs that were found in the diagnostic categories.

Weakness and contractures, frequent in neuromuscular disorders, did not generally occur in low-risk infants, including preterm infants at different gestational ages. While there are important maturational aspects in the quality of the movements that become more mature and fluent with increasing age, this does not affect the presence of antigravity movements. In preterm infants, antigravity movements were also always present even if their quality, with a higher rate of jerky abrupt movements, was different compared to term newborns. Contractures were also absent across the gestational age spectrum of low-risk infants. The module highlights the need to perfume a detailed assessment of contractures. While it is easy to identify contractures when there is a widespread pattern, as observed in arthrogryposis, other contractures, especially if localized to one or few joints, might be subtler and may be easily missed if not carefully evaluated by performing an assessment of the full range of extension of each joint.

The only item showing some variability with different gestation ages was the assessment of tendon reflexes, as some infants had tendon reflexes that were not easily elicitable with intermediate scores that were more frequent in term/late preterm newborns (17%) compared to preterm infants (4.7%). This observation is consistent with previous findings obtained in the validation of the general HNNE, showing that in full-term infants, reflexes can occasionally be more difficult to be elicited as opposed to preterm infants in whom tendon reflexes can be even exaggerated [13]. Tendon reflexes were therefore the only item in which some low-risk infants had scores in column two. These results suggest that not easily elicitable reflexes, when occurring in isolation, are not necessarily a negative prognostic sign in infants who do not have additional risk factors. The possibility to record on the same form information from prenatal and perinatal history was also found to be useful as a reminder to collect information that may increase the chance to identify additional diagnostic signs.

The application of the module to a cohort of floppy infants referred to our unit provided the opportunity to perform a small pilot study to assess the value of the module to detect and record correctly different signs. Although this study was not designed to assess the prognostic value of the new module as the cohort of floppy infants in whom it was piloted was very small, we still aimed to establish if the preliminary results were consistent with previous literature. We found that, in line with previous observations, weakness and contractures are the two signs that can more reliably identify infants with neuromuscular disorders [1, 2]. We confirmed that absent/extremely reduced antigravity movements were only found in patients with neuromuscular disorders who, in 90% of the cases, also showed contractures. Neonatal contractures are often the result of reduced motility in utero and can also be found in newborns with diagnoses of genetic etiologies or metabolic disorders [2, 4, 8]. Infants with other diagnoses in contrast had other abnormal signs but always had normal antigravity movements.

Our results suggest that the module can be easily used in a clinical setting as an add-on to the regular neonatal neurological examination in newborns identified as hypotonic on routine examination. The module is currently also used as an additional tool to the HNNE to identify possible early neurological signs in SMA presymptomatic patients identified through neonatal screening [14].

Even if, as recently suggested by a comprehensive study [7], new genetic techniques, including the combination of microarray karyotyping and exome sequencing may help in arriving at a diagnosis in the vast majority of infants with congenital hypotonia, we strongly believe that the diagnostic pathway should start from a detailed clinical examination to provide the first information guiding the choice of additional investigations and help in the differential diagnosis. Larger cohorts and multicentric studies are needed to establish the accuracy of the prognostic value of the module to identify other groups of conditions, such as genetic or metabolic disorders that were poorly represented in our cohort. Larger cohorts will also allow us to establish whether individual items can identify specific subgroups of neuromuscular disorders.

## Data Availability

All data are within manuscript; data are available upon reasonable request to the corresponding author.

## Abbreviations

CNS: Central nervous system
HNNE: Hammersmith Neonatal Neurological Examination
NGS: Next-generation sequencing

## References

[1] Dubowitz V (1968). The floppy infant–a practical approach to classification. Dev Med Child Neurol.

[2] Vasta I, Kinali M, Messina S, Guzzetta A, Kapellou O, Manzur A, Cowan F, Muntoni F, Mercuri E (2005). Can clinical signs identify newborns with neuromuscular disorders?. J Pediatr.

[3] Lisi EC, Cohn RD (2011). Genetic evaluation of the pediatric patient with hypotonia: perspective from a hypotonia specialty clinic and review of the literature. Dev Med Child Neurol.

[4] Prasad AN, Prasad C (2003). The floppy infant: contribution of genetic and metabolic disorders. Brain Dev.

[5] Laugel V, Cossee M, Matis J, de Saint-Martin A, Echaniz-Laguna A, Mandel JL, Astruc D, Fischbach M, Messer J (2008). Diagnostic approach to neonatal hypotonia: retrospective study on 144 neonates. Eur J Pediatr.

[6] Sparks SE (2015). Neonatal hypotonia. Clin Perinatol.

[7] Sharma S, Repnikova E, Noel-MacDonnell JR, LePichon JB (2021) Diagnostic yield of genetic testing in 324 infants with hypotonia. Clin Genet 10.1111/cge.14057 PMC9291145 34480364

[8] Mercuri E, Pera MC, Brogna C (2019). Neonatal hypotonia and neuromuscular conditions. Handb Clin Neurol.

[9] Savarese M, Di Fruscio G, Tasca G, Ruggiero L, Janssens S, De Bleecker J, Delpech M, Musumeci O, Toscano A, Angelini C, Sacconi S, Santoro L, Ricci E, Claes K, Politano L, Nigro V (2015). Next generation sequencing on patients with LGMD and nonspecific myopathies: findings associated with ANO5 mutations. Neuromuscul Disord.

[10] Savarese M, Di Fruscio G, Torella A, Fiorillo C, Magri F, Fanin M, Ruggiero L (2016). The genetic basis of undiagnosed muscular dystrophies and myopathies: Results from 504 patients. Neurology.

[11] Cummings BB, Marshall JL, Tukiainen T, Lek M, Donkervoort S, Foley AR, Bolduc V et al (2017) Improving genetic diagnosis in Mendelian disease with transcriptome sequencing. Sci Transl Med 9 10.1126/scitranslmed.aal5209 PMC5548421 28424332

[12] Ghaoui R, Cooper ST, Lek M, Jones K, Corbett A, Reddel SW, Needham M, Liang C, Waddell LB, Nicholson G, O'Grady G, Kaur S, Ong R, Davis M, Sue CM, Laing NG, North KN, MacArthur DG, Clarke NF (2015). Use of whole-exome sequencing for diagnosis of limb-girdle muscular dystrophy: outcomes and lessons learned. JAMA Neurol.

[13] Dubowitz L, Mercuri E, Dubowitz V (1998). An optimality score for the neurologic examination of the term newborn. J Pediatr.

[14] Pane M, Donati MA, Cutrona C, De Sanctis R, Pirinu M, Coratti G, Ricci M, Palermo C, Berti B, Leone D, Ticci C, Sacchini M, Cerboneschi M, Capasso A, Cicala G, Pera MC, Bravetti C, Abiusi E, Vaisfeld A, Vento G, Tiziano FD, Mercuri E (2022) Neurological assessment of newborns with spinal muscular atrophy identified through neonatal screening. Eur J Pediatr in press 10.1007/s00431-022-04470-3 PMC9192449 35522315

